# Mapping and validation of major quantitative trait loci for kernel length in wild barley (*Hordeum vulgare* ssp. *spontaneum*)

**DOI:** 10.1186/s12863-016-0438-6

**Published:** 2016-09-13

**Authors:** Hong Zhou, Shihang Liu, Yujiao Liu, Yaxi Liu, Jing You, Mei Deng, Jian Ma, Guangdeng Chen, Yuming Wei, Chunji Liu, Youliang Zheng

**Affiliations:** 1 Triticeae Research Institute, Sichuan Agricultural University, Wenjiang, Chengdu 611130 China; 2 CSIRO Agriculture Flagship, 306 Carmody Road, St. Lucia, QLD 4067 Australia

**Keywords:** Barley, Genetic linkage map, Kernel length, QTL, Validation, Candidate gene

## Abstract

**Background:**

Kernel length is an important target trait in barley (*Hordeum vulgare* L.) breeding programs. However, the number of known quantitative trait loci (QTLs) controlling kernel length is limited. In the present study, we aimed to identify major QTLs for kernel length, as well as putative candidate genes that might influence kernel length in wild barley.

**Results:**

A recombinant inbred line (RIL) population derived from the barley cultivar Baudin (*H. vulgare* ssp. *vulgare*) and the long-kernel wild barley genotype Awcs276 (*H.vulgare* ssp. *spontaneum*) was evaluated at one location over three years. A high-density genetic linkage map was constructed using 1,832 genome-wide diversity array technology (DArT) markers, spanning a total of 927.07 cM with an average interval of approximately 0.49 cM. Two major QTLs for kernel length, *LEN-3H* and *LEN-4H*, were detected across environments and further validated in a second RIL population derived from Fleet (*H. vulgare* ssp. *vulgare*) and Awcs276. In addition, a systematic search of public databases identified four candidate genes and four categories of proteins related to *LEN-3H* and *LEN-4H*.

**Conclusions:**

This study establishes a fundamental research platform for genomic studies and marker-assisted selection, since *LEN-3H* and *LEN-4H* could be used for accelerating progress in barley breeding programs that aim to improve kernel length.

**Electronic supplementary material:**

The online version of this article (doi:10.1186/s12863-016-0438-6) contains supplementary material, which is available to authorized users.

## Background

Barley (*Hordeum vulgare* L.) is one of the seven cereal crops grown worldwide and widely used in the animal feed and food industry. In 2012, barley was cultivated on 51.05 million hectares worldwide, resulting in the production of approximately 129.9 million metric tons (http://www.fao.org/home/en/). Barley is diploid (2n = 14), and its seven chromosomes share homology with those of other cereal species such as wheat, rye, and rice; therefore, it is an ideal species for genetic mapping and quantitative trait locus (QTL) analysis [1].

Significant progress has been made since the advent of molecular markers in genetic and QTL mapping. The first genetic map in barley was constructed using restriction fragment length polymorphism (RFLP) markers [2], whereas additional markers were used to build and improve barley linkage maps, including single nucleotide polymorphisms (SNPs), diversity array technology (DArT) markers, simple sequence repeats (SSRs), amplified fragment length polymorphisms (AFLPs), and sequence-tagged sites (STSs) [3–6]. Linkage maps enable general scientific discoveries, such as genome organization, QTL detection, and synteny establishment, whereas high-density maps are a useful tool in crop improvement programs to identify molecular markers linked to QTLs.

In barley, kernel length (LEN) is a major breeding target, since it is significantly correlated with grain yield. In previous studies, multiple QTLs for LEN have been fine-mapped. Ayoub et al. [7] reported a QTL for LEN in chromosome (Chr.) 3H; Backes et al. [8] reported two QTLs for LEN in Chr. 4H and 7H; Walker [9] detected QTLs for endosperm hardness, grain density, grain size, and malting quality using rapid phenotyping tools, and reported that 11 QTLs associated with LEN were significantly correlated with endosperm hardness, but not with grain density, using digital image analysis. Major QTLs for LEN have been also identified in rice, soybean [10], and wheat [11]. In rice, several loci associated with seed size and grain yield, including *GS3* [12], *GL7/GW7* [13], *qSW5/GW5* [14], *TGW6* [15], *An-1* [16], *BG2* [17], *OsSIZ1* [18], and *DST* [19], have been cloned through map-based cloning techniques. Of these, *An-1* encodes a bHLH protein and regulates awn development, kernel size, and kernel number [16]; *BG2* regulates kernel-related traits, including kernel thickness, kernel width, and thousand kernel weight [17]; *OsSIZ1* encodes E3 ubiquitin-protein ligases and regulates the vegetative growth and reproductive development [18]; and *DST* is a zinc finger transcription factor that regulates the expression of Gnla/OsCKX2 and improves grain yield [19].

In the present study, a recombinant inbred line (RIL) population derived from a cross between the barley cultivar Baudin (*H. vulgare* ssp. *vulgare*) and its wild relative Awcs276 (*H.vulgare* ssp. *spontaneum*) was evaluated in one location over three years in order to: (a) construct a high-density genetic linkage map using 1,832 DArT markers; (b) identify QTLs for LEN; (c) validate major QTLs for LEN in a second RIL population derived from a cross between Fleet (*H. vulgare* ssp. *vulgare*) and Awcs276; and (d) identify putative candidate genes that may influence LEN. Although many loci/QTLs for LEN have been identified previously in barley using marker-assisted selection, the discovery of additional loci/QTLs is necessary to enhance our understanding of the intricate genetic basis of kernel morphology and phenotype variance. These findings will provide new insights to improve barley yield in breeding programs.

## Methods

### RIL populations and phenotyping

**Fig. 1:**
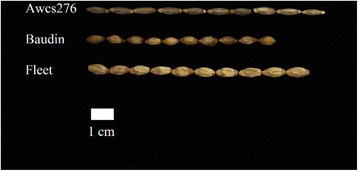
Kernel phenotypes of Awcs276, Baudin, and Fleet used for quantitative trait locus mapping in this study. Kernels in the upper line belong to the long-kernel parent Awcs276, those in the lower line belong to the short-kernel parent Fleet, and those in the middle line belong to the short-kernel parent Baudin

The spring barley cultivars Baudin and Fleet (*H. vulgare* ssp. *vulgare*) along with their wild relative Awcs276 (*H. vulgare* ssp. *spontaneum*) were obtained from a collection assembled at the University of Tasmania and used to generate two RIL populations (Fig. 1) as described by Chen [20]. Awcs276, a long-kernel wild barley genotype from the Middle East, was used as the common parent in the two RIL populations (Baudin/Awcs276 and Fleet/Awcs276). Baudin/Awcs276 (mapping population, 128 lines of F_8_, F_9_, and F_10_ generations) was evaluated in one location over three years to detect QTLs for LEN, whereas Fleet/Awcs276 (validation population, 94 lines of F_10_ generation) was evaluated for one year to validate putative QTLs identified in the mapping population. Baudin/Awcs276 was planted in October 2012 (F_8_), 2013 (F_9_), and 2014 (F_10_) in duplicate rows of ten plants each in a completely randomized design in Wenjiang, Chengdu, China (30°36′N, 103°41′E). The length of each row was 1.5 m with a row-to-row distance of 15 cm. Field management was carried out according to common practices in barley production. Mixed seeds were collected from mature plants in May 2013, 2014, and 2015, dried, and stored at 25 °C until analysis. Fleet/Awcs276 was planted in October 2014 and harvested in May 2015. Fully filled grains were used for measuring LEN in June 2015. LEN was measured in millimeters using a ruler and estimated by one measurement of 10 randomly selected kernels in 2013 or the average of three measurements in 2014 and 2015. The average LEN of each year was used for QTL analysis.

### Phenotypic data analysis

LEN in a given environment was determined as the arithmetic average of three biological replicates. Student’s *t*-test (*P* < 0.05) was used to identify the differences in LEN between the parental lines. Summary statistics were performed using Excel 2010 (Microsoft Corp., Redmond, WA, USA), whereas analysis of variance (ANOVA) in conjunction with Student’s *t*-test (*P <*0.001) using the general linear model (GLM) in SPSS 17.0 (IBM SPSS, Chicago, IL, USA). Broad-sense heritability (*H*^*2*^) for each trait was estimated as *H*^*2*^ = σ^2^_g_/(σ^2^_g_ + σ^2^_ge_/n + σ^2^_e_/nr), where σ^2^_g_ is the genetic variance, σ^2^_ge_ is the genotype by environment (G × E) variance, σ^2^_e_ is the error, n is the number of environments, and r is the number of replicates [21]. The σ^2^_g_, σ^2^_ge_, and σ^2^_e_ values were calculated using ANOVA (*P <*0.001) in SAS 9.2 (SAS Institute Inc., Cary, NC, USA). The best linear unbiased prediction (BLUP) method was used to estimate the random effects of mixed models. Phenotypic BLUP was calculated using the BLUP procedure in SAS 9.2.

### Genotyping and construction of genetic linkage map

Total genomic DNA (gDNA) was isolated and purified from fresh leaf tissue of one randomly selected plant in each F_8_ line of Baudin/Awcs276 and F_10_ line of Fleet/Awcs276 using the modified cetyltrimethylammonium bromide (CTAB) method [22]. DArT sequencing was conducted by Triticarte Pty Ltd. (Canberra, Australia), selecting the corresponding predominantly active genes of a genome fraction through the use of a combination of restriction enzymes, which separate low copy sequences from the repetitive fraction of the genome (http://www.diversityarrays.com/dart-application-dartseq). DArT sequencing generates two data types: 1) scores for “presence/absence” (dominant) markers, known as SilicoDArT markers, as they are analogous to microarray DArT markers, but are extracted *in silico* from sequences obtained from genomic representations; and 2) SNPs within the available genomic fragments. DArT loci were named according to their clone identification numbers as provided by Triticarte (http://www.diversityarrays.com/dart-application-dartseq-data-types). Polymorphic loci were selected from a total of 62,216 DArT markers after discarding those with a minor allele frequency of 0.4, a missing value of more than 20 %, or a common position.

The linkage map was constructed using IciMapping 3.2/4.0 [23] and JointMap4 [24]. All unanchored markers were properly grouped using IciMapping 3.2/4.0 with an LOD threshold of 3. The linkage analysis was conducted using JoinMap 4 (Kyazma, Wageningen, Netherlands) with a recombination frequency of 0.25, and all markers were grouped in the seven chromosomes.

### QTL mapping

Phenotypic data of each trait were the means of three biological replications in a single environment. The phenotypic BLUP was used to detect QTLs from the combined three-year data. QTL analysis for selected environments was performed through the interval mapping (IM) using MAPQTL6.0 (Kyazma, Wageningen, Netherlands) [25]. A test of 1,000 permutations was used to identify the LOD threshold that corresponds to a genome-wide false discovery rate of 5 % (*P* < 0.05). QTLs that were stable for a target trait across environments with clearly overlapping positions on the same chromosome were assumed to be the same. Stable QTLs that explained more than 10 % of the phenotypic variance for the specific trait were considered major QTLs [26].

QTLNetwork 2 [27] was used to determine QTLs with additive effects at individual loci, epistatic interactions between two different loci, and interactions between QTLs and the environment (QTL × E). The analysis was based on a mixed linear model (MLM) with 2 cM walking speed and 2D genome scan, which maps epistatic QTLs with or without single-locus effects using 1,000 permutations in order to generate a threshold for the presence of QTLs and QTL × E interactions.

### Marker development and QTL validation

Sequence information was obtained from the IPK Barley Blast Server (http://webblast.ipk-gatersleben.de/barley/index.php), and single-base differences were identified by high-resolution melt (HRM) analysis [28]. Markers were designed using Beacon Designer 7.9 and evaluated by Oligo 6.0 [29]. The parameters for Primer Premier (Premier Biosoft International, Palo Alto, CA, USA) were as follows: inner product size of 60–100 bp, melting temperature of 55 ± 5 °C, primer length of 20 ± 3 bp, and 3ʹ-end stability to avoid self-complementarity and primer dimer formation.

To detect markers, amplification reactions were performed in a total volume of 10 μl, containing 100 ng of template DNA, 5 μl of SsoFast EvaGreen mixture, 5 pmol of each forward and reverse primer, and DNase/RNase-free water up to the final value. PCR conditions were adjusted according to primer sets as follows: 4 min at 94 °C, 50 cycles of 1 s at 94 °C, and 30 s at 55 °C. This process is a precise warming of the amplicon DNA from approximately 65 °C to 95 °C. At some point during this process, the melting temperature of the amplicon is reached, and the two strands of DNA separate or “melt” apart [28].

The homozygous lines of Fleet/Awcs276 were used to validate major QTLs using the developed markers. Based on marker profiles, individuals were grouped into two classes: genotypes with homozygous alleles from AwcS276 and genotypes with homozygous alleles from Fleet. Student’s *t*-test (*P* < 0.05) was used to calculate the differences in LEN between these two classes of alleles and measure QTL effects within the validation population.

### Putative candidate gene identification

To identify putative coding gene regions, flanking candidate loci, or trait-related gene products, we used the corresponding QTL marker contigs to blast search against the WGSMorex database at the IPK Barley Blast Server (http://webblast.ipk-gatersleben.de/barley/index.php). We obtained QTL positions within the Morex reference map and putative trait-related proteins. According to the putative protein categories, most genes controlling kernel traits were identified in rice. The sequences of identified genes in rice were used to perform a BLASTN search against the barley database of the National Center for Biotechnology Information (NCBI, http://www.ncbi.nlm.nih.gov/) and the Phytozome website (https://phytozome.jgi.doe.gov/pz/portal.html) in order to identify homologous candidate genes in barley and other cereal crops.

## Results

### Phenotypic evaluation

The parental lines Awcs276 and Baudin showed significant differences in LEN (*P* <0.05) (Fig. 1, Additional file 1). The LEN (range, 7.12–7.97 mm; mean, 7.62 mm) of Awcs276 was higher than that of Baudin (range, 6.75–7.68 mm; mean, 7.28 mm). The trait variance over the three years and the phenotypic variance among RILs were high as shown by summary statistics, including range, mean, standard deviation, and coefficient of variation (Additional files 1, 2 and 3). The average LEN of 2013 was 8.11 mm (confidence interval, 8.011–8.192 mm), of 2014 was 7.25 mm (confidence interval, 7.185–7.313 mm), and of 2015 was 7.87 mm (confidence interval, 7.787–7.949 mm). The frequency of LEN and transgressive segregations were observed over the three years, indicating the presence of favorable alleles. The minimum LEN was 6.38 mm and the maximum 9.4 mm. The broad-sense heritability of LEN was low in 2013 (*h*^*2*^ = 0.122), owing to the lack of biological replications, high in 2014 (*h*^*2*^ = 0.937, F = 16.33, *P <* 0.0001) and 2015 (*h*^*2*^ = 0.870, F = 7.42, *P <* 0.0001), and moderate (*h*^*2*^ = 0.622, F = 11.5, *P <* 0.0001) over the three years, suggesting that genetic factors played an important role in the formation of LEN (Additional file 2). LEN showed normal or near-normal distribution with quantitative inheritance patterns suitable for QTL identification (Additional file 4).

### Genetic linkage map construction

A total of 1832 polymorphic markers (Additional file 5) was selected and mapped on eleven linkage groups (LGs) (Table 1, Additional file 6). The map spanned a total of 927.07 cM with an average marker distance of 0.49 cM. The results showed that Chr. 1H contained LG1 with a length of 133.31 cM, Chr. 2H contained LG2 and LG3 with a length of 261.6 cM, Chr. 3H contained LG4 and LG5 with a length of 116.05 cM, Chr. 4H contained LG6 with a length of 112.55 cM, Chr. 5H contained LG7 and LG8 with a length of 88.42 cM, Chr. 6H contained LG9 with a length of 93.21 cM, and Chr. 7H contained LG10 and LG 11 with a length of 121.92 cM. The largest LG was LG2, which contained 289 DArT markers, and the smallest was LG8, which contained only 87 markers. On average, each LG contained166.5 DArT markers and each Chr. contained 261.7 DArT markers. The genetic distances of the 11 LGs ranged from 22.10 cM (LG8) to 196.24 cM (LG2), and the average marker distance spanned from 0.25 cM (LG8) to 0.71 cM (LG1) (Table 1). Our genetic map was compared with other consensus maps [5] and the Morex reference map, and the results showed that the marker order had a satisfactory correspondence across the seven chromosomes.

**Table 1:** Basic information regarding the barley genetic map

Chr.	Linkage	Marker number	Map length (cM)	Marker interval (cM)
1H	LG1	188	133.31	0.71
2H	LG2	289	196.24	0.68
	LG3	109	65.36	0.60
3H	LG4	187	68.15	0.36
	LG5	135	47.90	0.35
4H	LG6	165	112.55	0.68
5H	LG7	129	66.32	0.51
	LG8	87	22.10	0.25
6H	LG9	230	93.21	0.41
7H	LG10	163	74.77	0.46
	LG11	150	47.15	0.31
Total		1832	927.07	0.49

*Chr* chromosome, *LG* linkage group, *cM* centimorgan

### QTL analysis and validation

**Fig. 2:**
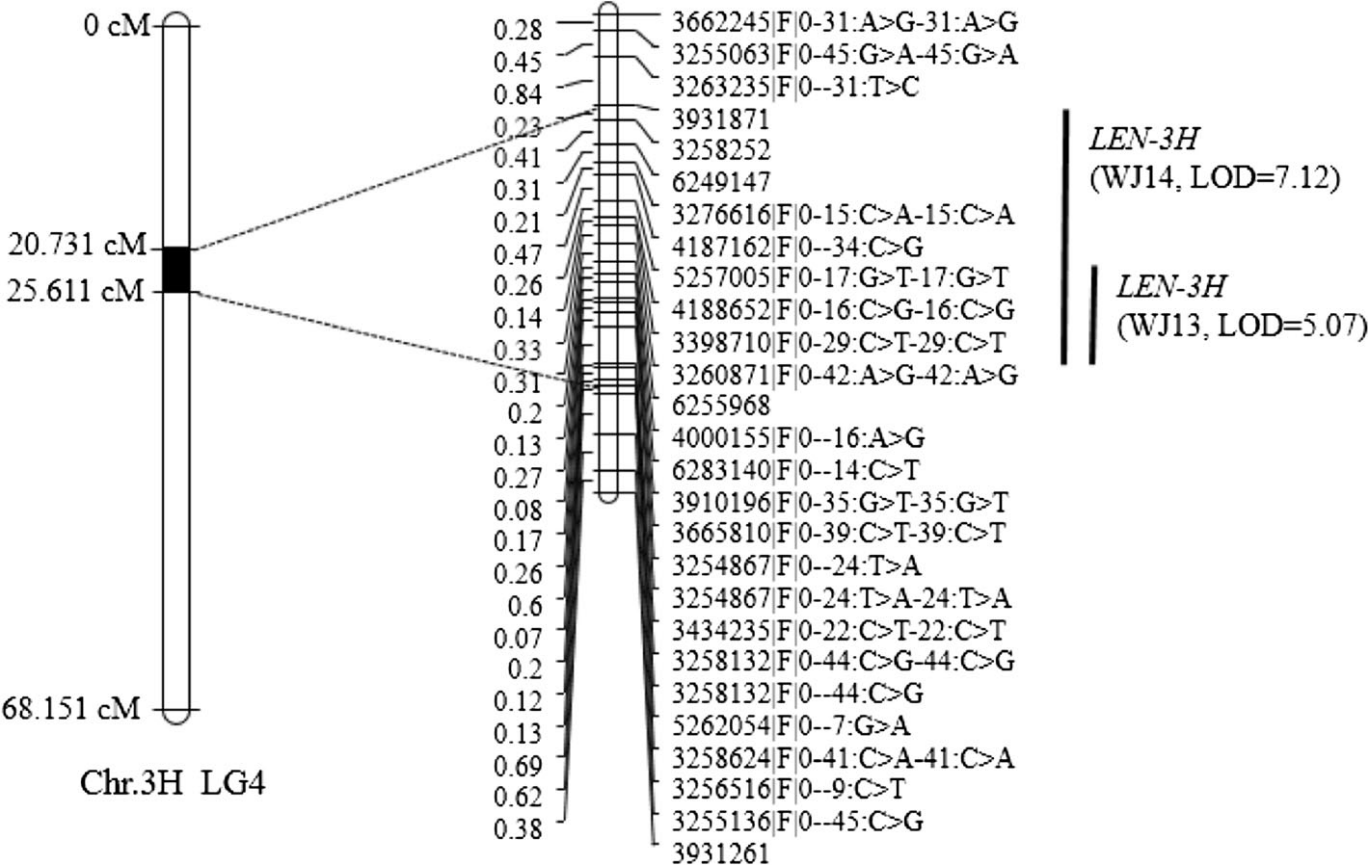
Linkage map of *LEN-3H* located on chromosome 3H, linkage group 4

**Fig. 3:**
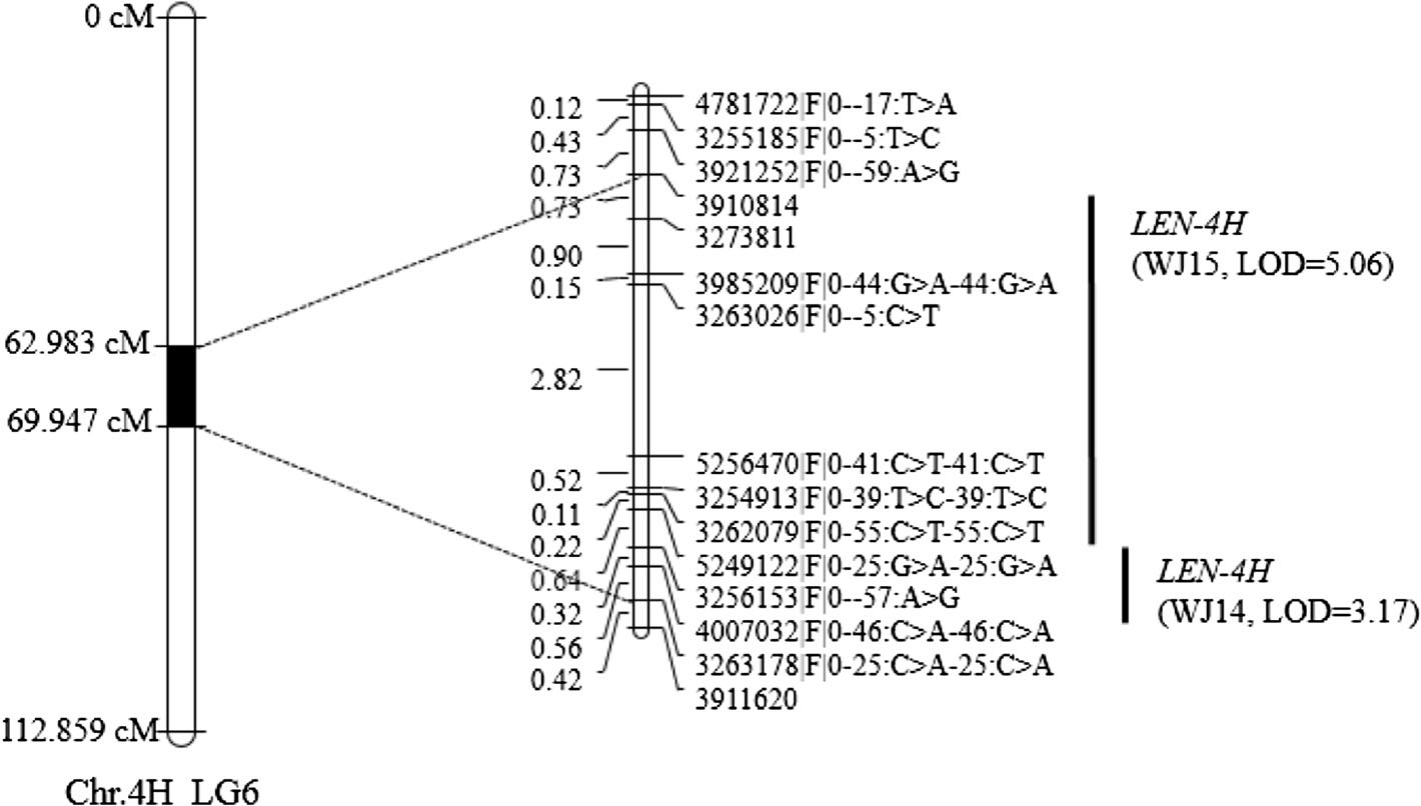
Linkage map of *LEN-4H* located on chromosome 4H, linkage group 6

Five significant QTLs were detected for LEN across the three environments (Table 2). The phenotypic variance explained by individual QTLs ranged from 10.4 % (*15LEN-2H*) to 29.1 % (*LEN-3H*). We used interval mapping for QTL analysis, and identified QTLs on all the chromosomes, except for 1H and 5H (Table 2). Two QTLs for LEN, *LEN-3H* and *LEN-4H*, were detected in different environments (Figs. 2 and 3); *LEN-3H* was identified in 2013 and 2014 and explained 29.1 and 22.3 % of the phenotypic variance, respectively, whereas *LEN-4H* was identified in different environments, having an LOD score of 3.17–5.06. Except for the two major QTLs, the rest three were environment-specific. Using BLUP, we identified four QTLs (*15LEN-2H, LEN-3H*, *LEN-4H*, and *14LEN-6H*) from the combined three-year data, all of which had positions similar to QTLs associated with the non-combined data. However, no QTLs were detected on 7H from the combined data (Table 2). Among the five QTLs for LEN, *LEN-3H* had additive main effects (a), whereas its interaction with the environment was not significant, showing high heritability (Table 3), whereas the rest four QTLs did not have additive effects.

**Table 2:** Quantitative trait loci (QTLs) for LEN identified in the Baudin/Awcs276 recombinant inbred line (RIL) population

QTL^a^	Chr.	Linkage	Environment	Left Marker	Right Marker	Range (cM)	LOD	% Expl.
*15LEN-2H*	2H	LG3	15WJ	3254852|F|0–65:C > A	6270031|F|0–48:C > G-48:C > G	16.326–17.508	3.11	10.4
			Combined	3254852|F|0–65:C > A	6270031|F|0–48:C > G-48:C > G	16.326-17.508	3.35	11.2
*LEN-3H*	3H	LG4	13WJ	6255968	3258624|F|0–41:C > A-41:C > A	23.405–25.611	5.07	29.1
			14WJ	3931871	3258624|F|0–41:C > A-41:C > A	20.731–25.611	7.12	22.3
			Combined	6249147	3258624|F|0–41:C > A-41:C > A	21.375–25.611	6.02	19.2
*LEN-4H*	4H	LG6	14WJ	5249122|F|0–25:G > A-25:G > A	3263178|F|0–25:C > A-25:C > A	68.431–69.947	3.17	10.6
			15WJ	3910814	5249122|F|0–25:G > A-25:G > A	62.983–68.431	5.06	16.4
			Combined	3396110	4007032|F|0–46:C > A-46:C > A	59.535-69.392	5.31	17.2
*14LEN-6H*	6H	LG9	14WJ	4594605|F|0–25:A > G-25:A > G	3259546|F|0–62:A > T-62:A > T	56.031–59.463	5.47	17.6
			Combined	4594605|F|0–25:A > G-25:A > G	3259546|F|0–62:A > T-62:A > T	56.031–59.463	3.92	13
*14LEN-7H*	7H	LG11	14WJ	3429688|F|0–38:T > C	3256863|F|0–29:G > A-29:G > A	19.095–22.504	5.31	17.2

*Chr* chromosome, *LG* linkage group, *cM* centimorgan, *Combined* combined data over the three years of study, % Expl the percentage of variance explained by QTL

^a^QTLs were identified by Interval Mapping (IM) using MAPQTL6.0, and a test of 1,000 permutations was used to identify the LOD threshold, corresponding to a genome-wide false discovery rate of 5 % (*P* < 0.05)

**Table 3:** Estimated additive and additive × environmental interactions of QTLs for kernel length (LEN) in barley

QTL name	Flanking interval	LOD	a effect^a^	ae1	ae2	ae3	QTL heritability				
							h^2^ (a)	h^2^ (ae)	h^2^ (ae1)	h^2^ (ae2)	h^2^ (ae3)
*LEN-3H*	23.4–25.6	7.12	−0.1599*	NS	NS	NS	0.1217	0.0139	0.0056	0.0027	0.0129

ae1, ae2, and ae3, QTL × environment interaction effect in 2013, 2014, and 2015, respectively

*NS* non-significant, *, significant at *P* < 0.001

^a^The analysis was based on a mixed linear model (MLM) with 1,000 permutations

The mixed linear model (MLM) was used to calculate the estimated additive (a) and additive × environment interactions (ae)

Based on the sequences of tightly linked DArT markers, we BLAST-searched against the Ensembl Barley database at the Ensembl Plants Blast Server (http://plants.ensembl.org) and found that *LEN-3H* was located on Chr. 3HL, whereas *LEN-4H* on Chr. 4HL. Next, we BLAST-searched the sequences of tightly linked DArT markers against the Morex reference map database and converted DArT markers to HRM markers for tracking QTLs using quantitative real-time PCR. Accordingly, two primer pairs were designed and used to track *LEN-3H* and *LEN-4H* (Additional file 7).

In this study, two major QTLs were validated in Fleet/Awcs276 (Table 4). For *LEN-3H*, the average LEN of genotypes with homozygous alleles from Awcs276 was significantly higher (*P* < 0.05) than that of genotypes with homozygous alleles from Fleet. Similarly, for *LEN-4H*, the average LEN of genotypes with homozygous alleles from Awcs276 was significantly higher (*P* < 0.05) than that of genotypes with homozygous alleles from Fleet. Detailed information is presented in Additional files 8 and 9.

**Table 4:** Validation of two quantitative trait loci (QTLs) in the Fleet/Awcs276 recombinant inbred line (RIL) population

QTL	Chr.	AA	BB	*P* value^a^
*LEN-3H*	3H	8.79	9.05	0.01**
*LEN-4H*	4H	8.83	9.03	0.03*

*AA* homozygous alleles from Fleet, *BB* homozygous alleles from Awcs276, *Chr* chromosome

^a^Student’s t-test (*P* < 0.05) was used to identify differences between the parental lines; **, significant at *P* < 0.01; *, significant at *P* < 0.05

### Putative candidate genes

For the two major QTLs for LEN in Baudin/Awcs276, we found several putative candidate genes for kernel-related traits, and these genes could be divided into four categories (Table 5): the first category included genes related to defense response such as salt tolerance; the second category included genes related to receptors such as ethylene receptors; the third category included genes related to transcription factors and promoters such as basic helix-loop-helix (bHLH) DNA-binding superfamily proteins and MADS-box transcription factors; and the fourth category included genes related to various enzymes such as zinc finger CCCH domain-containing proteins, E3 ubiquitin-protein ligases, and cytochrome P450.

**Table 5:** Putative genes or proteins of major quantitative loci (QTLs) for kernel length in barley

Stable QTLs	Chr.	Putative candidate genes	Gene in rice	Putative genes in barley	*Zea mays*	*Arabidopsis thaliana*	*Brachypodium distachyon*	*Panicum hallii*	*Sorghum bicolor*
*LEN-3H*	3H	Zinc finger CCCH domain-containing protein	*DST*	*AK365156.1*	*GRMZM2G089448*	*AT4G33660*	*Bradi1g06420*	*Pahal.I01451*	*Sobic.001G065500*
		E3 ubiquitin-protein ligase BRE1-like protein	*OsSIZ1*	*AK366345.1*	*GRMZM2G155123*	*AT5G60410*	*Bradi2g38030*	*Pahal.C01170*	*Sobic.009G026500*
		Cytochrome P450	*GE; CYP78A13; BG2*	*AK374135.1*	*GRMZM2G138008*	*AT1G74110*	*Bradi4g35890*	*Pahal.B03875*	*Sobic.002G367600*
		Polyglutamine-binding protein 1							
		Ankyrin-repeat protein							
		FeS assembly protein							
		Calcium-dependent protein kinase							
*LEN-4H*	4H	Basic helix-loop-helix (bHLH) DNA-binding Superfamily protein	*An-1*	*AK361814.1*	*GRMZM5G828396*	*AT4G36540*	*Bradi5g06620*	*Pahal.G01160*	*Sobic.001G105000*
		Salt tolerant-related protein							
		LEA hydroxyproline-rich glycoprotein family							
		Seed maturation protein PM41							
		MADS-box transcription factor 1							
		Ethylene receptor							

*Chr* chromosome

## Discussion

Awcs276 is a long-kernel wild barley genotype that has been previously used in genetic studies, because of its relatively long seeds, extensive environmental adaption, and high genetic diversity that can provide abundant germplasm resources for genetic variation and crop improvement [20, 30, 31]. Awcs276 was used in the present study owing to its having genes that are superior for LEN to those of the Australian barley cultivars Baudin and Fleet. Therefore, two RIL populations were developed by crossing Awcs276 with Baudin and Fleet to identify QTLs for LEN. Two major QTLs (*LEN-3H* and *LEN-4H*) were identified from Awcs276 in two environments. *LEN-3H* was detected in 2013 and 2014 in the interval of 20.731–25.611 cM on Chr. 3H using MAPQTL6.0. A peak within this interval was also identified in 2015 with a maximum LOD of 1.19, explaining 4.1 % of the phenotypic variance (Additional file 10). Both the environmental variation and G × E interaction were highly significant (*P* < 0.0001) (Additional file 2). These results showed that the environment influenced the QTLs, explaining the reason that none QTL was found in all the experimental years. The effects of *LEN-3H* and *LEN-4H* were evaluated in Fleet/Awcs276, and the results showed that these two QTLs stably increase LEN in barley.

A QTL for kernel length was identified between 55.8 cM and 84.3 cM on Chr. 3H in a previous study [7]. Furthermore, five markers (ABG462, PSR156a, ABG453, ABG499, and M351316) were found within this interval, and information on the marker ABG453 was obtained from GrainGenes (http://wheat.pw.usda.gov/GG3/). Therefore, we used the parental lines and some extreme phenotypes in their progenies to confirm ABG453, and found that it was polymorphic for the parental lines. Backes et al. [8] reported a QTL for kernel length on Chr. 4H in an interval of 12 cM and identified four markers (MWG2033, MWG0857, MWG0611, and MWG0921) within it. In the present study, we found the nearby loci of MWG2033 in the Hv-Consensus2006-Marcel-4H from GrainGenes and used the parental lines to confirm the nearby markers. The marker HVM40 was polymorphic for the parental lines with a distance of 4.1 cM from MWG2033 in the consensus map. Thus, ABG453 and HVM40 were used for genotyping the lines of Baudin/Awcs276 (Additional file 11). Next, we used these two markers along with DArT markers to construct a genetic map and found that ABG453 (69.142 cM) and HVM40 (95.841 cM) were mapped on LG4 and LG6, respectively (Additional file 12). Using BLUP, we identified *LEN-3H* and *LEN-4H* in the interval of 20.428–25.917 cM and 59.02–69.119 cM, respectively. ABG453 (69.142 cM) and HVM40 (95.841 cM) were not included in the QTL interval, thus we speculated that the QTLs detected by Ayoub et al. [7] and Backes et al. [8] were not the same as *LEN-3H* and *LEN-4H.* In general, the two QTLs for kernel size that were identified in this study were within a relatively small interval, which makes them an ideal target for breeding programs as well as for the characterization of gene(s) underlying this locus.

Kernel size is a major determinant of grain weight and an important yield component [32]. It refers to the space bounded by the husks, measured by LEN and width, and serves as a component of grain yield that determines kernel weight [33]. LEN was an important trait for barley domestication and has been a major target in barley breeding, because of its direct influence on grain yield. In the present study, according to four categories of putative proteins that influence LEN and several homologous candidate genes in *Zea mays*, *Arabidopsis thaliana*, *Brachypodium distachyon*, *Panicum hallii*, and *Sorghum bicolor*, we identified four putative candidate genes (NCBI accession no. AK361814.1, AK365156.1, AK366345.1, and AK374135.1) (Table 5). The putative candidate gene (NCBI accession no. AK361814.1) for *LEN-4H* was homologous to *An-1* in rice. And *An-1* encodes a bHLH protein that positively regulates cell division, grain length, and awn elongation, but negatively regulates the grain number per panicle in rice [16]. The other three putative candidate genes (NCBI accession no. AK365156.1, AK366345.1, and AK374135.1) for *LEN-3H* were homologous to *DST*, *OsSIZ1*, and *BG2*, respectively (Table 5). *DST* is a zinc finger transcription factor that improves grain yield and regulates the expression of Gnla/OsCKX2 [19]. Li et al. [34] reported that *DSTreg1* enhances panicle branching and increases the grain number. And *OsSIZ1* encodes E3 ubiquitin-protein ligases that regulate the growth and development in rice [18]. Wang et al. [35] reported that *ossiz1* mutants have shorter primary and adventitious roots than wild-type plants, suggesting that *OsSIZ1* is associated with the regulation of root architecture and acts as a regulator of the Pi (N)-dependent responses in rice. *BG2* encodes *OsCYP78A13*, which has a paralog in rice (*Grain Length 3.2; GL3.2, LOC_Os03g30420*) with distinct expression patterns [17]. *CYP78A13* is highly expressed in seeds at 5–8 day after planting, whereas *GL3.2* is specifically expressed in the roots [17]. Analysis of transgenic plants harboring either *CYP78A13* or *GL3.2* revealed that both genes can promote grain growth by positively affecting LEN, kernel thickness, kernel width, and thousand kernel weight [17]. Overall, all the four genes control seed length or grain yield in rice, and the corresponding proteins are the putative candidate proteins of *LEN-3H* and *LEN-4H*. Hence, the two major QTLs, *LEN-3H* and *LEN-4H*, and the four putative candidate genes might play crucial and dynamic roles in the control of LEN in barley and other grain crops.

## Conclusion

In this study, we identified two major QTLs for LEN (*LEN-3H* and *LEN-4H*) derived from Baudin/Awcs276 and validated in Fleet/Awcs276. Additionally, four putative candidate genes that might control LEN and four categories of putative proteins that might have a phenotypic effect were identified for the two major QTLs. The QTLs and putative candidate genes identified in this study provide important information for barley genetic studies and breeding programs.

## Additional files

Additional file 1: Phenotypic performance of barley kernel length of Baudin/Awcs276 recombinant inbred lines (RILs) population. (XLSX 11 kb) Additional file 2: Analysis of variance (ANOVA) for kernel length of the Baudin/Awcs276 recombinant inbred line (RILs) population over the three years. (XLSX 10 kb) Additional file 3: Average kernel length of the Baudin/Awcs276 recombinant inbred line (RILs) population. (XLSX 14 kb) Additional file 4: Frequency distributions of kernel length in the Baudin/Awcs276 recombinant inbred line (RILs) population over the three years. (XLSX 26 kb) Additional file 5: Genotyping information of the Baudin/Awcs276 recombinant inbred line (RILs) population. (XLSX 1007 kb) Additional file 6: Linkage maps constructed using the Baudin/Awcs276 recombinant inbred line (RILs) population. (XLSX 60 kb) Additional file 7: Information for high-resolution melt (HRM) markers developed based on the linkage genome-wide diversity array technology (DArT) markers. (XLSX 10 kb) Additional file 8: Average kernel length of the Fleet/Awcs276 recombinant inbred line (RILs) population. (XLSX 11 kb) Additional file 9: Genotyping information of the Fleet/Awcs276 recombinant inbred line (RILs) population. (XLSX 12 kb) Additional file 10: Information regarding the peak marker within the interval of *LEN-3H* detected in 2015.. (XLSX 14 kb) Additional file 11: Genotyping of the Baudin/Awcs276 recombinant inbred line (RILs) population using the markers ABG453 and HVM40. (XLSX 12 kb) Additional file 12: Mapping positions of the markers ABG453 and HVM40 on linkage group (LG) 4 and LG6. (XLSX 20 kb)

## Abbreviations

ANOVA: Analysis of variance
BLUP: Best linear unbiased prediction
Chr: Chromosome
cM: Centimorgan
DArT: Genome-wide diversity array technology
HRM: High-resolution melt
IM: Interval mapping
LEN: 10-Kernel length
LG: Linkage group
MLM: Mixed linear model
QTL: Quantitative trait locus
RIL: Recombinant inbred line
SNP: Single nucleotide polymorphism
SSR: Single sequence repeat
